# Dissemination of metaldehyde catabolic pathways is driven by mobile genetic elements in Proteobacteria

**DOI:** 10.1099/mgen.0.000881

**Published:** 2022-10-27

**Authors:** Víctor Castro-Gutierrez, Edward Fuller, María Pilar Garcillán-Barcia, Thorunn Helgason, Francis Hassard, James Moir

**Affiliations:** ^1^​ Department of Biology, University of York, Heslington, York, UK; ^2^​ Cranfield University, College Road, Cranfield, Bedfordshire, MK43 0AL, UK; ^3^​ Environmental Pollution Research Center (CICA), University of Costa Rica, Montes de Oca, 11501, Costa Rica; ^4^​ Instituto de Biomedicina y Biotecnología de Cantabria, Universidad de Cantabria-Consejo Superior de Investigaciones Científicas, Santander, Cantabria, Spain

**Keywords:** bioremediation, evolution, insertion sequence, metaldehyde, pesticides, plasmid

## Abstract

Bioremediation of metaldehyde from drinking water using metaldehyde-degrading strains has recently emerged as a promising alternative. Whole-genome sequencing was used to obtain full genomes for metaldehyde degraders *Acinetobacter calcoaceticus* E1 and *Sphingobium* CMET-H. For the former, the genetic context of the metaldehyde-degrading genes had not been explored, while for the latter, none of the degrading genes themselves had been identified. In *A. calcoaceticus* E1, IS*91* and IS*6*-family insertion sequences (ISs) were found surrounding the metaldehyde-degrading gene cluster located in plasmid pAME76. This cluster was located in closely-related plasmids and associated to identical ISs in most metaldehyde-degrading β- and γ-Proteobacteria, indicating horizontal gene transfer (HGT). For *Sphingobium* CMET-H, sequence analysis suggested a phytanoyl-CoA family oxygenase as a metaldehyde-degrading gene candidate due to its close homology to a previously identified metaldehyde-degrading gene known as *mahX*. Heterologous gene expression in *Escherichia coli* alongside degradation tests verified its functional significance and the degrading gene homolog was henceforth called *mahS*. It was found that *mahS* is hosted within the conjugative plasmid pSM1 and its genetic context suggested a crossover between the metaldehyde and acetoin degradation pathways. Here, specific replicons and ISs responsible for maintaining and dispersing metaldehyde-degrading genes in α, β and γ-Proteobacteria through HGT were identified and described. In addition, a homologous gene implicated in the first step of metaldehyde utilisation in an α-Proteobacteria was uncovered. Insights into specific steps of this possible degradation pathway are provided.

## Data Summary

Short-read and long-read sequences have been deposited in the European Nucleotide Archive (ENA) (http://www.ebi.ac.uk/ena) under BioProject ID PRJEB40574 for *A. calcoaceticus* E1 and under BioProject ID PRJEB51438 for *Sphingobium* CMET-H. All strains are available from the authors upon request.

**Impact Statement**

Pollution of drinking water is a global consequence of pesticide use. The development of effective and low-cost technologies for its treatment is needed for use in both developing countries and in industrialized nations. Metaldehyde has frequently been detected in drinking water at levels above the regulatory limits as a consequence of diffuse pesticide pollution, and bioremediation strategies have been effective at eliminating this compound at pilot-scale in the short term. Nevertheless, several knowledge gaps still exist regarding its biological degradation. The mechanisms for dispersion of metaldehyde-degrading genes in β- and γ-Proteobacteria have not been uncovered, and no metaldehyde-degrading proteins have been identified in α-Proteobacteria. An in-depth analysis of the mobile genetic elements involved in the horizontal transfer of the metaldehyde-degrading trait in α-, β- and γ-Proteobacteria is provided, showing that insertion sequences and catabolic plasmids are central to its dissemination amongst the studied prokaryotes. Additionally, through homology analysis, we identified the genetic determinant for the initial step of metaldehyde degradation in *Sphingobium* CMET-H (α-Proteobacteria) and explored specific steps of a possible degradation pathway. A complete understanding the underlying processes that mediate the transmission of metaldehyde degradation capabilities will allow more elaborate bioremediation strategies to be pursued in drinking water biological purification systems in the near future.

## Introduction

Metaldehyde is a molluscicide pesticide used to control slugs and snails in agricultural crops and domestic gardens which has been found to pollute water resources and result in compliance failures in drinking water [1–3]. Bioremediation of metaldehyde from drinking water using metaldehyde-degrading strains has emerged as a promising alternative for its treatment through bioaugmentation, which refers to the addition of exogenous microbial populations or communities with proven pollutant depuration capabilities to a contaminated system, has been used for this purpose [4, 5].

**Fig. 1.**
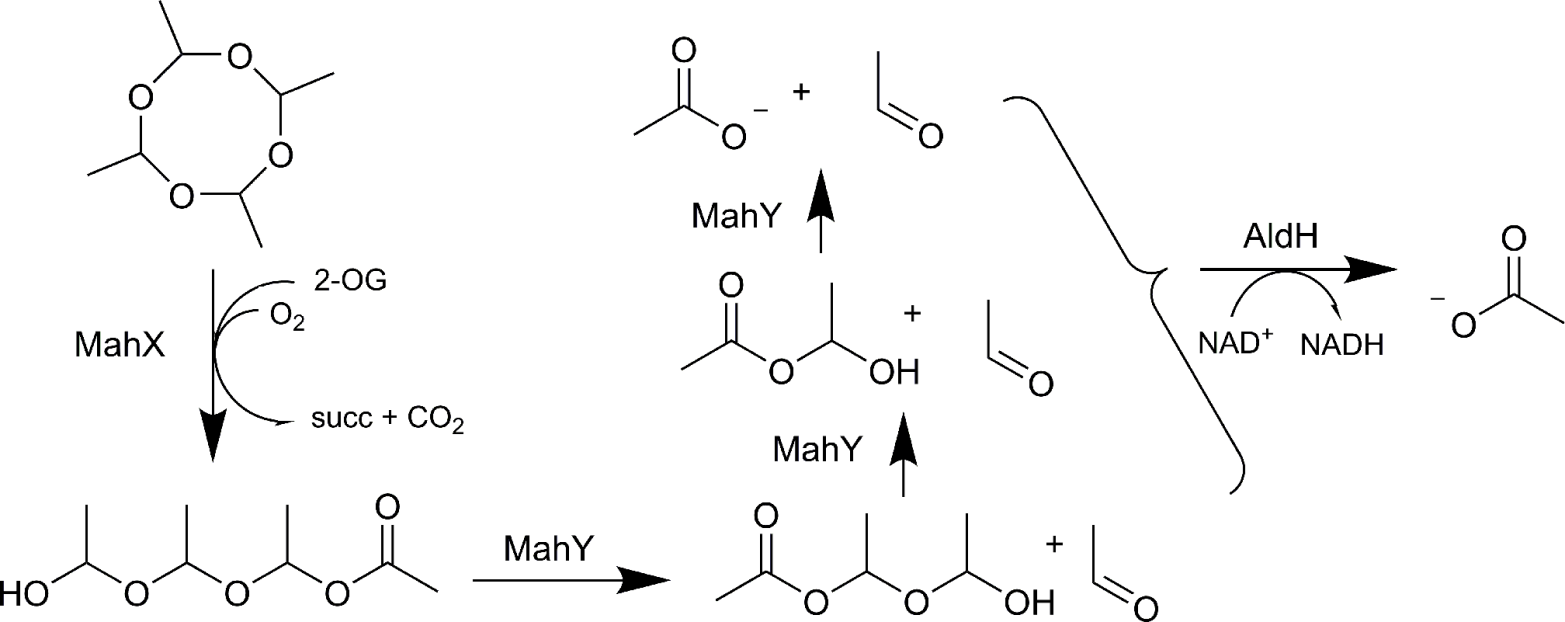
Predicted pathway for metaldehyde degradation [8]. MahX is related to 2-oxoglutarate (2-OG)-dependent oxygenases that generate succinate (succ) and CO_2_. MahX oxygenates metaldehyde to release a linear hemiacetal that is cleaved iteratively into acetaldehyde +a shorter chain hemiacetal, and eventually acetate. AldH oxidises acetaldehyde to acetate in an NAD^+^-dependent reaction.

Isolation of metaldehyde-degrading strains began to be explored by Thomas *et al.* [6] who were the first to isolate metaldehyde-degrading strains, namely *A. calcoaceticus* E1 and *Variovorax* E3. A study by Castro-Gutiérrez *et al.* [7] subsequently expanded the number and diversity of identified degraders by isolating two additional *Acinetobacter* strains, as well as strains from the genera *Pseudomonas *, *Caballeronia *, *Sphingobium* and *Rhodococcus.* Identification of a cluster of genes shared by strains of *Acinetobacter, Pseudomonas* and *Caballeronia* led to the recognition of the MahX protein as being responsible for the first step of metaldehyde transformation. A degradation pathway was proposed based on the activity of the products of the *mahX* gene and the contiguous genes in the cluster, *mahY* and *aldH* (Fig. 1). Furthermore, quantitative polymerase chain reaction (qPCR) analyses indicated that peaks in the amount of *mahX a*nd *mahY g*enes in soil coincided with metaldehyde removal in 50 % of the soils tested, supporting the environmental relevance of this pathway [8].

The high similarity of metaldehyde-degrading protein sequences between isolates in β and γ-Proteobacteria suggests that horizontal gene transfer (HGT) through mobile genetic elements (MGEs) may mediate the dissemination of this trait [7]. MGEs refer to genetic elements which promote intracellular or intercellular DNA mobility. In bacteria, examples of the former include transposons, insertion sequences and integrons, while the latter are usually represented by conjugative or mobilizable plasmids, integrative conjugative elements, and bacteriophages [9]. The determinants responsible for the degradation of organic xenobiotics in bacteria have often been associated with MGEs, for example for compounds such as toluene, parathion or atrazine [10–15]; however, their involvement in the biological degrading pathways for metaldehyde is still unknown.

Third generation long-range sequencing technologies have been used to gain insights into xenobiotic-degrading prokaryotes for compounds such as linuron [16], dibenzofuran [17], 2,6-dichlorobenzamide [18] and organophosphorus insecticide [19] degraders, and can also be applied to explore MGEs for metaldehyde. In-depth knowledge of MGEs and HGT mechanisms for this compound can open new possibilities for drinking water bioremediation. For instance, genetic bioaugmentation, the stimulation of horizontal transfer of catabolic plasmids between exogenous donor cells and indigenous bacteria [20] could be applied to confer metaldehyde-degrading capabilities to microorganisms that already inhabit biological water purification systems such as slow sand filters in the event that conjugative plasmids are unmistakably identified within degrading strains.

For metaldehyde degrading isolates within taxa other than β and γ-Proteobacteria (* Variovorax *, *Rhodococcus* and *Sphingobium *), genes responsible for metaldehyde degradation and their mechanisms of dispersion remain undescribed. Metaldehyde degrader *Sphingobium* CMET-H has been shown to deplete environmental-significant concentrations of metaldehyde (2.0 µg l^−1^) to undetectable levels in real waters in bench- and pilot-scale slow sand filters [5]. Identification of the genes and enzymes which are involved in metaldehyde degrading pathways and their HGT mechanisms in this and other strains could allow for the development of effective, cheap and environmentally-friendly biotechnological applications.

Here, we set out to explore the genetic context of metaldehyde degrading genes in α, β, and γ-Proteobacteria. Specific plasmids and insertion sequences (ISs) responsible for maintaining and dispersing metaldehyde-degrading genes in *Acinetobacter *, *Pseudomonas *, *Caballeronia *, and *Sphingobium* strains were identified and described, a homologous gene implicated in metaldehyde utilisation in an α-Proteobacteria (* Sphingobium *) was uncovered, and insights into specific steps of a possible degradation pathway are provided.

## Methods

### Combined Illumina / Oxford Nanopore sequencing for selected metaldehyde degraders

Combined Illumina and Oxford Nanopore sequencing was undertaken with the aim to obtain the complete and fully assembled genome sequence for the reference metaldehyde degrader *Acinetobacter calcoaceticus* E1 [6], and for the novel degrader *Sphingobium* CMET-H [7], a strain shown capable of removing metaldehyde at micropollutant levels from drinking water [5]. Short read Illumina MiSeq sequencing for other metaldehyde degraders: *Acinetobacter bohemicus* JMET-C, *Acinetobacter lwoffii* SMET-C, *Pseudomonas vancouverensis* SMET-B, and *Caballeronia jiangsuensis* SNO-D had been reported previously [7].

Each strain was grown to late exponential phase in LB broth (30 °C, 150 r.p.m.), cells were gently pelleted down (10 min, 500 ***g***), supernatant was removed, cells were resuspended in cryopreservative fluid using wide bore filter tips and stored in a provided Microbank bead tube (Pro-Lab Diagnostics, United Kingdom). The sequencing service was provided by MicrobesNG (United Kingdom). For the Illumina short-read sequencing, three beads were washed with extraction buffer containing lysozyme and RNase A and incubated for 25 min at 37 °C. Subsequently, proteinase K and RNaseA were added and incubated for 5 min at 65 °C. Genomic DNA was purified using an equal volume of solid phase reversible immobilization beads and resuspended in elution buffer. DNA was quantified in triplicates with the Quantit dsDNA HS assay (Invitrogen, UK) in an AF2200 (Eppendorf, Germany) plate reader. Genomic DNA libraries were prepared using Nextera XT Library Prep Kit (Illumina, USA) following the manufacturer’s protocol with the following modifications: two nanograms of DNA instead of one were used as input, and PCR elongation time was increased to 1 min. DNA quantification and library preparation were carried out on a Microlab STAR automated liquid handling system (Hamilton, USA). Pooled libraries were quantified using the Kapa Biosystems Library Quantification Kit for Illumina (Roche, South Africa) on a Roche light cycler 96 qPCR machine. Libraries were sequenced on the Illumina HiSeq using a 250 bp paired end protocol. Reads were adapter trimmed using Trimmomatic 0.30 with a sliding window quality cut-off of Q15 [21]. For Oxford Nanopore sequencing, approximately 2×10^9^ cells were used for high molecular weight DNA extraction using Nanobind CCB Big DNA Kit (Circulomics, USA). DNA was quantified with the Qubit dsDNA HS assay in a Qubit 3.0 fluorometer (Invitrogen, United Kingdom). Long read genomic DNA libraries were prepared with Oxford Nanopore SQK-RBK004 kit and SQK-LSK109 kit with Native Barcoding EXP-NBD104/114 (ONT, United Kingdom) using 400–500 ng of HMW DNA. Twelve to twenty-four barcoded samples were pooled together into a single sequencing library and loaded in a FLO-MIN106 (R.9.4) flow cell in a GridION (Oxford Nanopore Technologies, United Kingdom).

### Bioinformatic analyses of whole-genome sequencing data

*De novo* genome assembly was performed using Unicycler v0.4.0 [22]. Assembly graphs were inspected using Bandage v0.8.1 [23] and contigs were annotated using Prokka v1.11 [24]. PlasmidSPAdes v3.14.0 [25] was used to determine the putative gene composition and structure of plasmids from short read DNA sequencing only. Additional functional annotations were performed using eggNOG-mapper against the EggNOG V 5.0 database [26, 27] and the PATRIC annotation service [28]. Replication origin location was determined using the DoriC replication origin database [29]. GC content and GC skew were determined using Artemis v18.1.0 [30]. rRNA operons were identified using RNAmmer v1.2 [31]. tRNA genes were identified using tRNAscan-SE [32]. The origin of transfer (*oriT*) and conjugation-related regions were determined using the OriT finder server [33]. MOBscan [34] was used to detect and classify relaxases. The type IV coupling proteins (T4CPs) and type IV secretion systems (T4SSs), which constitute the mating-pair formation system (MPF), were detected and classified using MacSyFinder [35]. Plasmids were analysed with COPLA [36] to determine their taxonomic unit. Searches against the ISFinder database [37] were used to characterize transposases and ISs. Protein comparison between multiple sequences was performed and associated plots were generated using the PATRIC Proteome Comparison Service [28]. The Burrows-Wheeler Alignment tool (BWA) in UGENE v40.1 was used to map short DNA sequencing reads against specific scaffolds. InterPro [38] was used to predict protein families and domains. Single sequence nucleotide and protein comparisons were carried out using nBLAST or pBLAST searches [39]. Plots were generated using DNAplotter v18.1.0 [40]. PlasmidSeeker [41] was used to estimate the plasmid copy number (PCN) of the plasmids present in *Acinetobacter calcoaceticus* E1 and *Sphingobium* CMET-H from their Illumina reads. The plasmid sequences from these strains were used to build PlasmidSeeker databases and their chromosome sequences were used as reference genomes. Illumina MiSeq reads for the other metaldehyde degraders, *Acinetobacter bohemicus* JMET-C, *Acinetobacter lwoffii* SMET-C, *Pseudomonas vancouverensis* SMET-B, and *Caballeronia jiangsuensis* SNO-D had been previously deposited in the European Nucleotide Archive under study PRJEB30540 [7].

### Identification of putative metaldehyde-degrading determinants in *Sphingobium* CMET-H

To identify potential metaldehyde degrading proteins in the *Sphingobium* CMET-H predicted proteome, tBLASTn [42] was used to align the metaldehyde degrading MahX and MahY protein sequences from *A. calcoaceticus* E1 [7] to the *Sphingobium* CMET-H genome translated in all frames. Candidates were excluded if related (≥30 % identity) proteins were present in the predicted proteome of *Sphingobium chlorophenolicum* NBRC 16172 (NCBI accession 1219044) a closely related strain which does not have the ability to degrade metaldehyde [43].

### Phylogenetic analysis of plasmid-encoded phytanoyl-CoA dioxygenase (PhyH) family proteins

The proteins MahX from plasmid pAME76 and MahS from plasmid pSM1 were used as queries in a hmmscan search against the pfam database using default parameters [44]. Members of the pfam PhyH family, to which MahX and MahS belong, encoded in the RefSeq200 plasmid database were retrieved with the hmmsearch function of HMMER 3.1b2 (--incE 0.001 –incdomE 0.001) [45]⁠ using the PhyH Hidden Markov Model (HMM) profile PF05721.16. The proteins were aligned with MAFFT v7.310 [46]⁠. The alignment was trimmed with trimAl 1.2rev59 (-gt 0.8 -resoverlap 0.75 -seqoverlap 90) [47]. ModelFinder [48] was used to predict the best-fit evolutionary model, according to the Bayesian information criterion. Using the LG+F+R6 model, a maximum-likelihood phylogenetic reconstruction was inferred with IQ-TREE version 1.6.1 [49]⁠. Branch support was estimated by performing 1000 ultrafast bootstrap (UFBoot) approximation replicates [50]. The resulting tree was visualized and annotated using the online tool iTOL v4 [51]⁠.

### Heterologous expression of the putative metaldehyde-degrading gene *mahS*

Heterologous expression of putative metaldehyde degrading gene *mahS* from *Sphingobium* CMET-H was carried out in *E. coli* to validate the hypothesis that its product catalyses the first step of metaldehyde degradation. Briefly, the *mahS* gene was amplified from *Sphingobium* CMET-H genomic DNA using primers mbp-mahS F and mbp-mahS R, and inserted into plasmid pETFPP_2 [52], generating a *mahS* construct with an N-terminal His tag and a maltose binding protein tag under IPTG inducible expression (PT7 promoter) and carrying kanamycin resistance. *E. coli* BL21(DE3) was transformed with the vector and subsequently cultured in Luria Bertani broth. Protein expression was corroborated in SDS-PAGE gels [53]. Expression of MBP-MahS was induced. Subsequently, metaldehyde was added to a final concentration of 15 mg l^−1^. *E. coli* BL21(DE3) with an empty pETFPP_2 vector was used as control. All assays were performed in triplicate. After a 3.5 h incubation, metaldehyde was quantified using gas chromatography as described below. Details are included in Supplementary Methods - Heterologous expression of *mahS* and Table S1.

### Metaldehyde extraction and quantification

Aqueous samples were centrifuged (4000 ***g***, 10 min) and 0.4 ml supernatant added to 0.5 ml dichloromethane in glass chromatography vials (Thermo Fisher Scientific), vortexed for 30 s and stabilized for 20 min. Then 5 µl of organic phase were injected into an Agilent 7820A gas chromatograph (Stockport, UK) fitted with a HP-5 column and Flame Ionization Detector. Chromatographic parameters were described previously [54]. Limit of detection and limit of quantification were 0.05 mg l^−1^ and 0.15 mg l^−1^ respectively. Calibration curves were constructed with standard metaldehyde solutions in minimal salts media (MSM) [6] and sample peak area was interpolated in the calibration curves.

## Results

### Whole-genome sequencing for *A. calcoaceticus* E1

Illumina short-read sequencing and Oxford Nanopore sequencing were used in combination to provide a better understanding of the genomic organization of metaldehyde degrader *A. calcoaceticus* E1. Quality statistics for Illumina short-read sequencing and preliminary assembly are shown in Table S2. Oxford Nanopore sequencing (mean coverage was 51.8×) provided the long reads needed to be used as a scaffold for the Illumina sequencing data. The complete and fully assembled genome sequence was thus successfully obtained and consists of a circular chromosome and two circular plasmids (Table 1 and Fig. S1). Detailed description of the replicons is provided in Supplementary Results – Technical description of the *A. calcoaceticus* E1 genome.

**Table 1.** Resulting contigs from combined Illumina and Oxford Nanopore sequencing and assembly of metaldehyde-degrading strains *A. calcoaceticus* E1 and *Sphingobium* CMET-H

Strain	Replicon	Length (bp)	Type of replicon	Topology	GC content (%)	tRNA	rRNA
***Acinetobacter*** ***calcoaceticus* E1**	Chr. 1	4 406 004	Chromosome	Circular	38.8	75	6
	pAME76	76 942	Plasmid	Circular	41.9	0	0
	pAC10	10 549	Plasmid	Circular	40.5	0	0
	Total	4 493 495	–	–	38.8	75	6
*** Sphingobium *** **CMET-H**	Chr. 1	3 632 600	Chromosome	N.D.*	63.9	51	1
	Chr. 2	1 706 133	Chromosome	Circular	63.3	4	2
	pSM1	150 281	Plasmid	Circular	62	0	0
	pSM2	60 892	Plasmid	Circular	60.5	0	0
	pSM3	15 107	Plasmid	Circular	63.6	0	0
	Total	5 565 013	–	–	63.6	55	3

### Metaldehyde-degrading genes are located in plasmid pAME76 in *A. calcoaceticus* E1

Annotation identified 81 CDSs in the 76.9 kb circular plasmid (Fig. S1), from here on called pAME76, of which three are the metaldehyde-degrading proteins. The identified plasmid replication initiation protein identified belongs to the Rep_3 superfamily (PF01051). No *oriT* region, relaxase gene, T4CP and T4SS components were found in the plasmid which implies that pAME76 is non-transmissible by conjugation. pAME76 is a low copy-number plasmid (2.8 copies per chromosome).

**Fig. 2.**
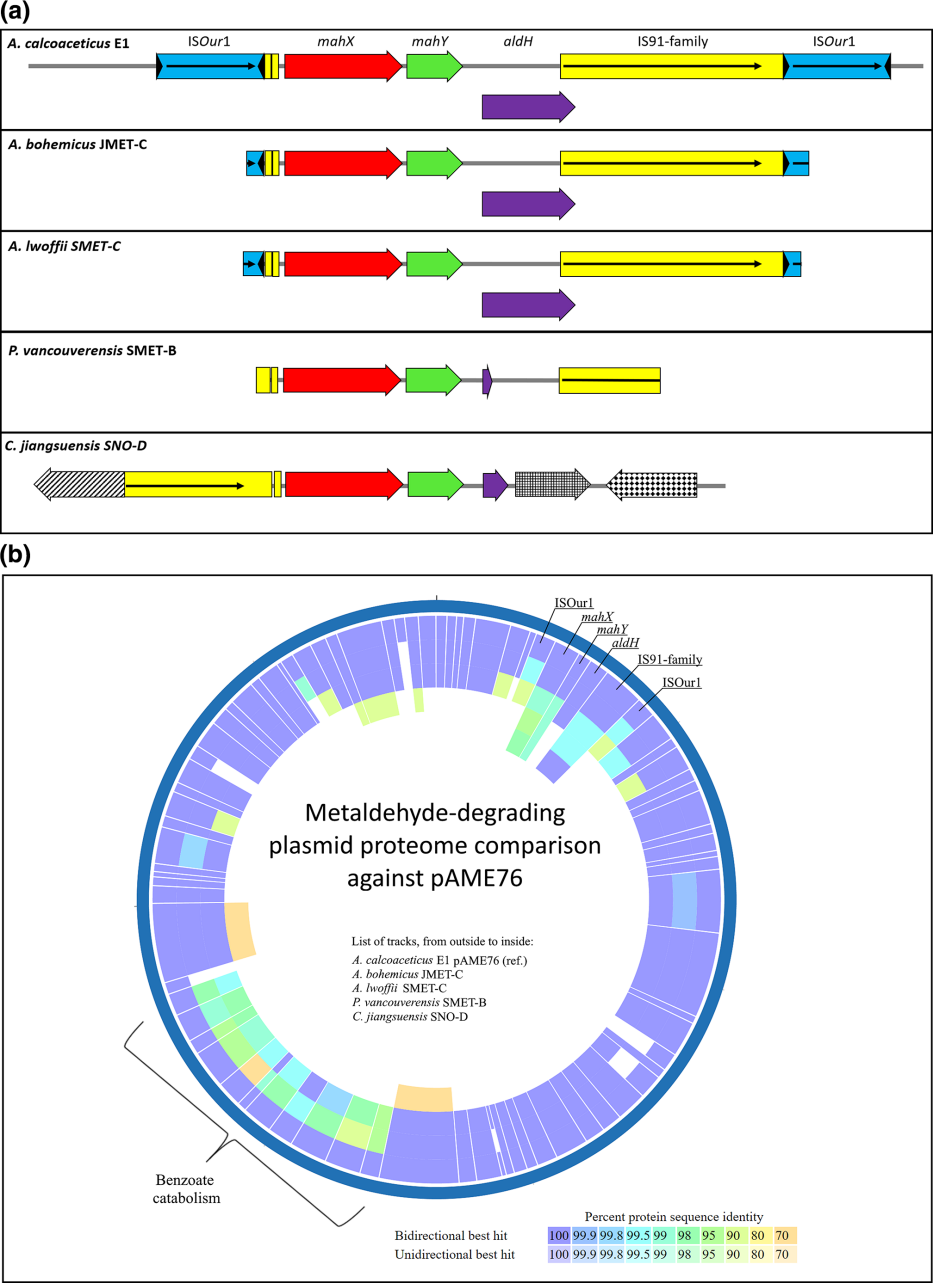
a. Immediate genetic context of metaldehyde degradation genes in metaldehyde-degrading strains that share the same initial pathway. Black triangles represent terminal inverted repeats. Black horizontal arrows represent transposase ORFs. Gene and IS names are indicated for *A. calcoaceticus* E1 and colour coded for the rest of the strains. For *C. jiangsuensis* SNO-D tilted line pattern indicates a partial IS*21* transposase (96.7 % identity; PKO63873.1); mesh pattern indicates a predicted thiolase family protein and tile pattern indicates a sequence related to sensor histidine kinase proteins. Gene maps not to scale.** b.** Diagram of proteins encoded by pAME76 and presence of similar sequences in metaldehyde-degrading strains that share the same degradation pathway. The analysis was performed using PATRIC Proteome Comparison Service [28]. Minimum cut-off values of 70 % identity and 40 % coverage were established.

The three previously identified genes from the metaldehyde-degrading cluster (*mahX*, *mahY* and *aldH*) and an IS*91* family transposase are flanked by two IS*6* family transposases in bases 4975 to 10474 (Fig. 2a). Upon closer inspection using ISFinder [37] it was determined that these IS*6*-like elements are two identical copies of an intact IS*Our*1 insertion sequence first described in *Oligella uretralis* (coordinates 268–1087 of GenBank Acc. no. AY177427, >99 % identity, 820 bp) [55] in direct orientation, probably comprising a composite transposon. The GC content for the section comprising the degrading genes and the inner IS*91* transposase is 62.1 %, considerably higher than the average GC content of the plasmid (41.9 %) or the chromosome (38.8 %), suggesting a relatively recent acquisition from a different taxon [56].

The COPLA analysis of this plasmid showed that it is not closely related to any plasmid assigned to a plasmid taxonomic unit (PTU). nBLAST search of the plasmid sequence revealed that a section of the plasmid (bases 39746 to 53607) is 98.8 % similar to plasmid pXG03-X3 from *Acinetobacter indicus* strain XG03 (CP045128.1). This region includes a series of genes responsible for benzoate catabolism [57] flanked by two ISAcsp1 transposases in opposite orientation (100.0 % similarity, 2967 bp). Another section of the plasmid with higher GC content than the average (bases 72807 to 76150) includes a series of genes involved in mercuric resistance that includes *merD*, *merA*, *merC* and *merR* genes found in numerous plasmids from *Acinetobacter* species [58].

### Genetic context of the metaldehyde-degrading gene cluster in *A. calcoaceticus* E1

The complete sequence of plasmid pAME76 from *A. calcoaceticus* E1 can be used as a reference to compare the genetic context of the metaldehyde-degrading genes in the rest of the isolates that share the same initial degradation pathway (Fig. 2a). In this strain the metaldehyde-degrading genes (*mahX*, *mahY, aldH*) and a partial IS*91* family insertion sequence with a truncated origin and lacking the terminal section (1827–1863 and 1–111 bp missing respectively vs. Genbank accession AH010393.2) are flanked by two intact copies of the IS*Our*1 insertion sequence. Also, two 45 bp partial repeated end fractions of a similar IS*91* family insertion sequence are present upstream of the degrading genes. The sequence of the *aldH* gene has been interrupted by the insertion of the IS*91* family element, resulting in an open reading frame containing both, the partial *aldH* gene and a fraction of the IS that extends until a stop codon is reached (231 aa; original length 506 aa WP_133879878.1).

### Genetic context of the metaldehyde-degrading gene cluster in the other degrading strains

Although in the other two *Acinetobacter* strains (* A. bohemicus* JMET-C and *A. lwoffii* SMET-C) the comparison is limited by the size of the contig obtained through Illumina sequencing, it is shown that end fractions of the IS*Our*1 copies also surround the metaldehyde-degrading cluster and the IS*91*-like elements in these strains (Fig. 2a). In fact, the 4307 bp contig from *A. bohemicus* JMET-C is identical to the corresponding section from *A. calcoaceticus* E1. Meanwhile the 4229 bp contig from *A. lwoffii* SMET-C is 99.97 % similar to these two. These two observations further suggest the horizontal transfer of the degrading cluster and the IS*91* internal copy through the action of the flanking IS*Our*1 elements in the *Acinetobacter* strains.

In both, *P. vancouverensis* SMET-B and *C. jiangsuensis* SNO-D the *mahX* and *mahY* genes are present upstream from truncated *aldH* genes (Fig. 2a). In the case of strain SMET-B, no IS*Our*1 copies are present within the contig nor in the whole-genome sequencing data. Here, a mutation in the *aldH* gene has generated a premature stop codon in the sequence, rendering only a 12 amino acid open reading frame for the gene. In strain SNO-D, IS*Ou*r1 is also absent in the contig as well as in the genome. A 1174 bp fraction of the IS*91* element with the exact same sequence as the one present in *A. calcoaceticus* E1 is, in this case, located upstream from the degrading gene cluster instead of downstream. It does not contain the full transposase gene, the origin is truncated in the same way its counterpart in *A. calcoaceticus* E1 is, and it lacks 694 bp in the terminal section. In this case the *aldH* gene is also truncated by a premature stop codon generating a 99 amino acid open reading frame.

### Metaldehyde-degrading genes are contained in putative pAME76-like plasmids in *A. bohemicus* JMET-C and *A. lwoffii* SMET-C

To explore the possibility that the same or a similar plasmid harbours the metaldehyde-degrading operon in other strains, a search and comparison of the proteins encoded by pAME76 in the other metaldehyde-degrading isolates was undertaken (Fig. 2b). The absence of most of the pAME76 plasmid encoded proteins for degraders *P. vancouverensis* SMET-B and *C. jiangsuensis* SNO-D (innermost rings) revealed that in these isolates the metaldehyde-degrading operon is not located in a pAME76-like plasmid. On the other hand, most of the proteins in pAME76 have homologs in *A. bohemicus* JMET-C and *A. lwoffii* SMET-C, including an identical *repB* replication initiation protein (Rep_3 superfamily) in both, indicating that they may contain at least one plasmid with a similar replication machinery that could be harbouring the degrading operon. However, for the genes involved in benzoate compound catabolism, the similarity of the homologs present in these other two strains was lower, which indicates that they do not contain plasmids with an identical sequence to pAME76. Mapping the reads from the whole genome sequencing of *A. bohemicus* JMET-C and *A. lwoffii* SMET-C against the pAME76 revealed two prominent drops in the read mapping coverage of the pAME76 plasmid scaffold for both isolates (Fig. S2). The first one (26 to 28 kbp position approx.) corresponded to the absence of a *lexA* repressor in both degrading isolates, whilst the second one (42 to 53 kbp position approx.) indicated a much lower coverage of the benzoate catabolic region when compared to the rest of the plasmid. In contrast, read mapping coverage for the metaldehyde-degrading gene operon (5.8 to 8.1 kb position approx.) was relatively high.

Sequencing coverage for genes located in plasmids is usually higher than the coverage for those located in the chromosome, owing to the usually higher copy number of plasmids in prokaryotic genomes [59, 60]. These data suggested that in strains *A. bohemicus* JMET-C and *A. lwoffii* SMET-C the benzoate-degrading pathway is chromosomally encoded and, more importantly, that the metaldehyde-degrading gene operon is probably contained in plasmids in each of these two isolates, as it is in strain *A. calcoaceticus* E1. Nonetheless, these plasmids are not identical to pAME76, although they have many CDSs in common.

The putative gene composition of the plasmids present in strains *A. bohemicus* JMET-C and *A. lwoffii* SMET-C was predicted using the short-read Illumina whole genome sequencing data as input in PlasmidSPAdes [25]. For *A. bohemicus* JMET-C, 22 contigs (112515 bp) were assigned to a putative plasmid containing 114 CDS including the metaldehyde-degrading operon, the mercury resistance genes and a *repB* protein identical to that of pAME76. For *A. lwoffii* SMET-C, 28 contigs (105139 bp) were assigned to a putative plasmid containing 124 CDS also containing the metaldehyde-degrading operon, the mercury resistance genes and an identical *repB* protein (Fig. S3). Both putative plasmids encode MOB_Q_ relaxase proteins, but no MPF components, and thus are potentially mobilizable. They both lack the benzoate catabolic genes and do not belong to assigned PTUs. This analysis supports the idea that metaldehyde-degrading genes are also located in plasmids in strains JMET-C and SMET-C.

The location of the metaldehyde-degrading operon in *P. vancouverensis* SMET-B and *C. jiangsuensis* SNO-D was also assessed using this approach. For strain SMET-B, four contigs (71187 bp) were assigned to a putative plasmid containing 62 CDSs including the metaldehyde-degrading operon, a MOB_P_ relaxase gene, a T4CP and T4SS of the MPF_T_ type, suggesting that it is a conjugative plasmid. For strain SNO-D, the metaldehyde degrading operon was not predicted to be found in a plasmid; hence, it would be either chromosomally encoded or part of a plasmid with a copy number close to one.

### Whole-genome sequencing for *Sphingobium* CMET-H

Illumina short-read sequencing and Oxford Nanopore sequencing were used in combination to investigate the genomic organization of metaldehyde degrader *Sphingobium* CMET-H. Quality statistics for Illumina short-read sequencing and preliminary assembly are shown in Table S2. Oxford Nanopore sequencing (mean coverage was 59.8×) provided the long reads to be used as a scaffold for the Illumina sequencing data. The genome consists of two chromosomes and three circular plasmids (Table 1 and Fig. S4). Circular topology was identified for all the replicons except for Chromosome 1 (seven contigs), for which repetitive regions prevented a definitive resolution, nevertheless, inspection of the assembly graph using Bandage [23] revealed that all these contigs belong to the same molecule.

### A large putatively conjugative plasmid is found in the *Sphingobium* CMET-H genome

**Fig. 3.**
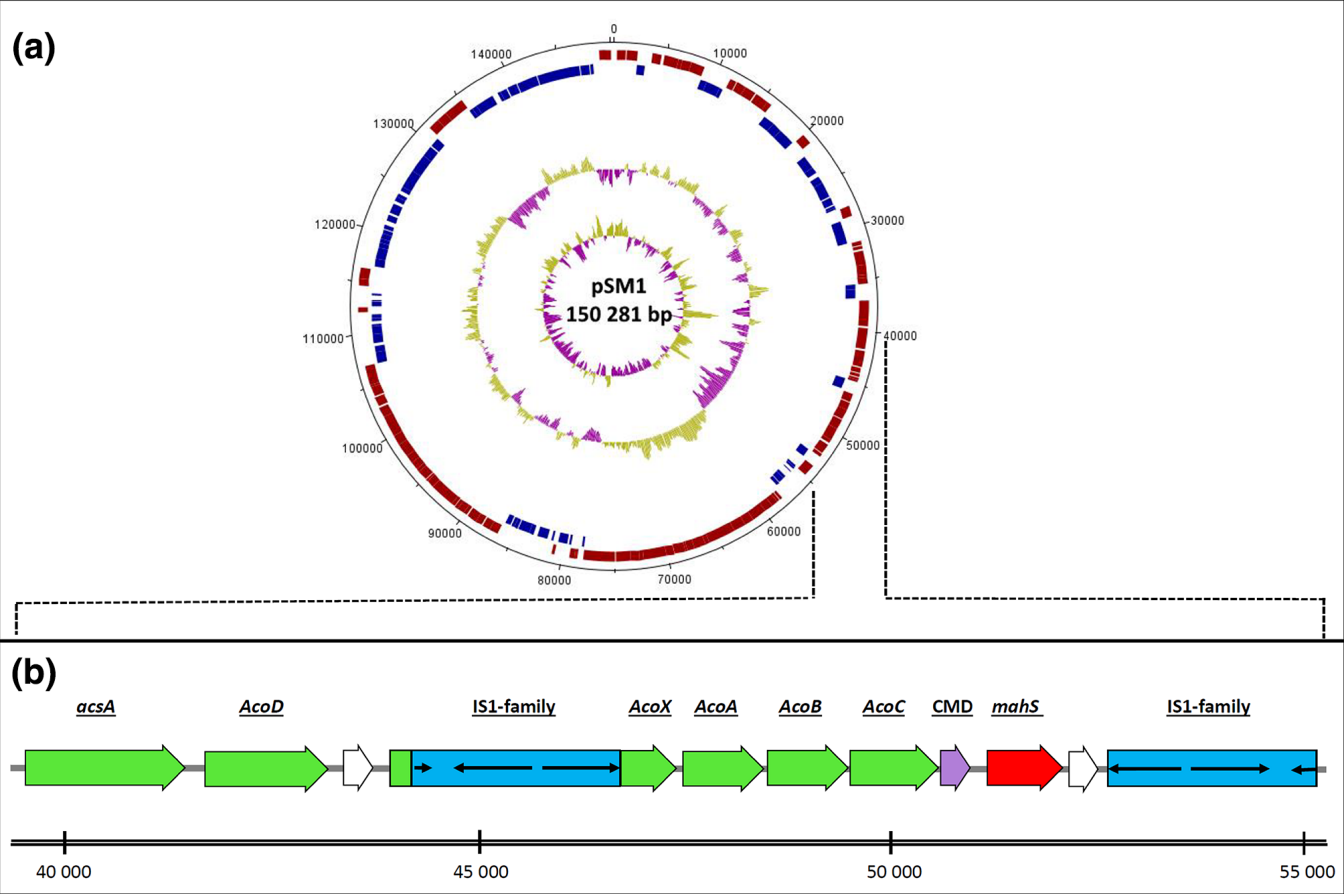
a. Plasmid pSM1 from *Sphingobium* CMET-H; from outside to inside: open reading frames for the forward and reverse strands in red and blue; GC content (purple: below average, gold: above average); GC skew (purple: below average, gold: above average). **b.** Immediate genetic context of metaldehyde degrading gene *mahS* (red), with a carboxymuconolactone decarboxylase family protein gene (CMD, purple), genes coding for enzymes implicated in acetoin metabolism (green) and IS*1*-family insertion sequences (blue).

PROKKA annotation identified 163 CDSs in a 150.3 kb circular low copy-number plasmid (PCN=1.49), from now on called pSM1 (Fig. 3a), of which 32 are related with conjugative transfer, including a MOB_P_ class relaxase, a T4CP and two T4SSs corresponding to the MPF_T_ and MPF_F_ types, which indicates that pSM1 is a conjugative plasmid. Other CDSs identified encoded 10 transposases or inactive derivatives, seven related to acetoin metabolism, seven involved in plasmid replication and partitioning (including a Rep_3 replication initiation protein), one phytanoyl-CoA dioxygenase family protein, and the rest encode for various other functions of have no annotation.

### A close homolog to the MahX protein was found in the *Sphingobium* CMET-H genome

To identify potential homologues of the metaldehyde-degrading MahX protein from *A. calcoaceticus* E1 within *Sphingobium* CMET-H, the MahX amino acid sequence was used with tBLASTn to query the *Sphingobium* CMET-H genome. The alignment sequence with the highest score possessed a 57 % identity, and a query coverage of 87 %. The identified protein, henceforth known as MahS (encoded by the *mahS* gene), consists of 307 aa residues. blast conserved putative domain analysis on MahS identified domains which belong to the αKG -Fe (II) oxygenase superfamily, between the residues 40–236. InterPro and Pfam analyses revealed that, similar to MahX, MahS contains domains which belong to the phytanoyl-CoA dioxygenase family PhyH (IPR008775, PF05721).

**Fig. 4.**
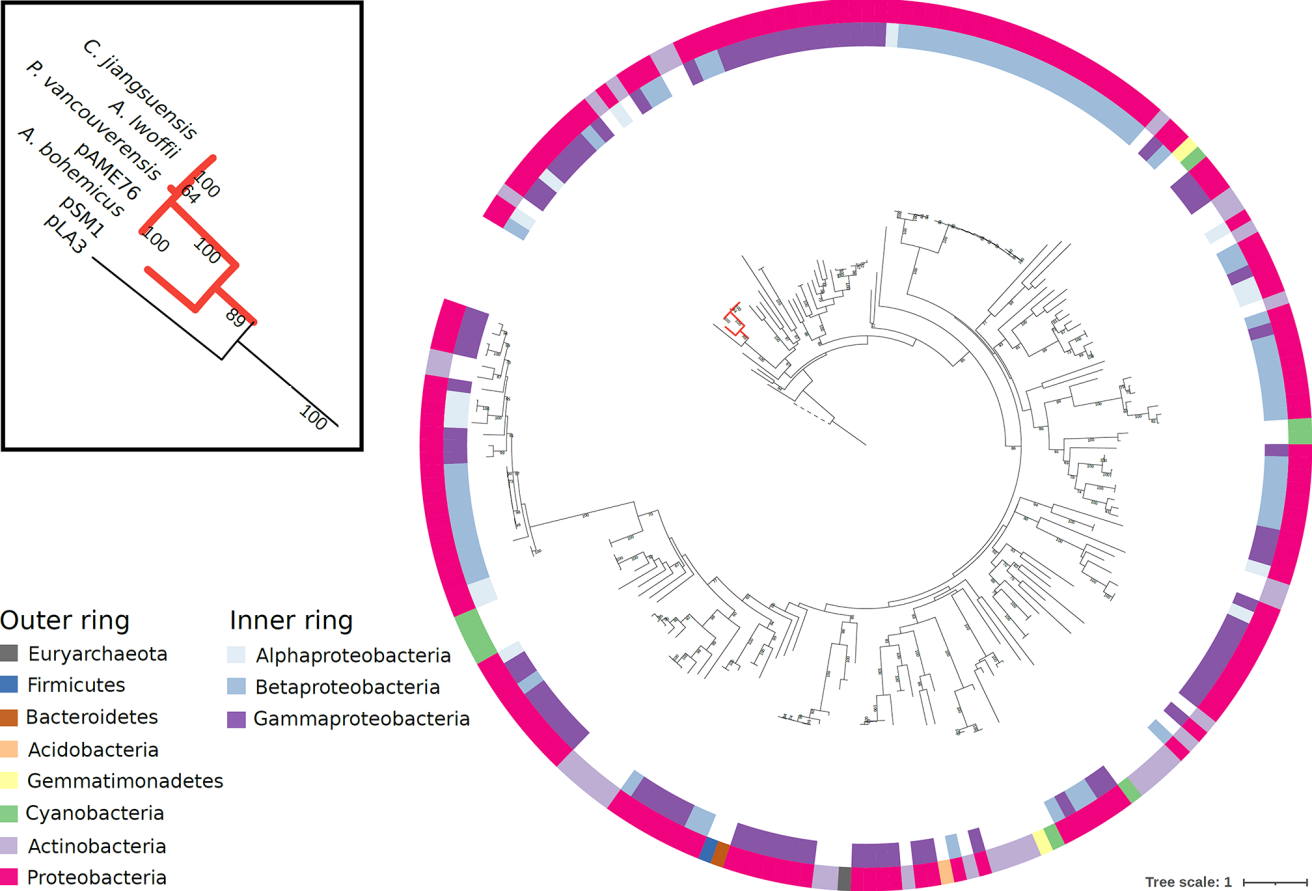
Phylogenetic tree of plasmid-encoded PhyH homologs. The maximum-likelihood tree was constructed with 208 PhyH homologs encoded in plasmids. The YJR154W PhyH homolog of *Saccharomyces cerevisiae* (UniProt identifier P47181, included in the PF05721.16 seed alignment) was used as an outgroup for rooting the tree, and its branch is shown as a discontinuous line. UFBoot support values >60 % are shown. The clade containing the PhyH homologs included in this study is highlighted in red and zoomed in the inset at the left of the figure. The outer ring indicates the phylum of the plasmid bacterial host. The inner ring indicates the taxonomic class when the plasmid is present in Proteobacteria.

Homologs belonging to the PhyH family are present in many prokaryotic plasmids, mainly from hosts of the bacterial phylum Proteobacteria (Fig. 4). As shown in the phylogenetic tree, the PhyH proteins encoded in plasmids are highly divergent. The MahX homologs studied here (the plasmids from *Acinetobacter* and *Pseudomonas* strains, and the putatively chromosome-encoded homolog from *C. jiangsuensis* SNO-D) are highly similar and cluster together in a well-supported clade with MahS of pSM1. The short evolutionary distance between the MahX homologs suggests that they have been recently transferred between different genomic platforms.

**Fig. 5.**
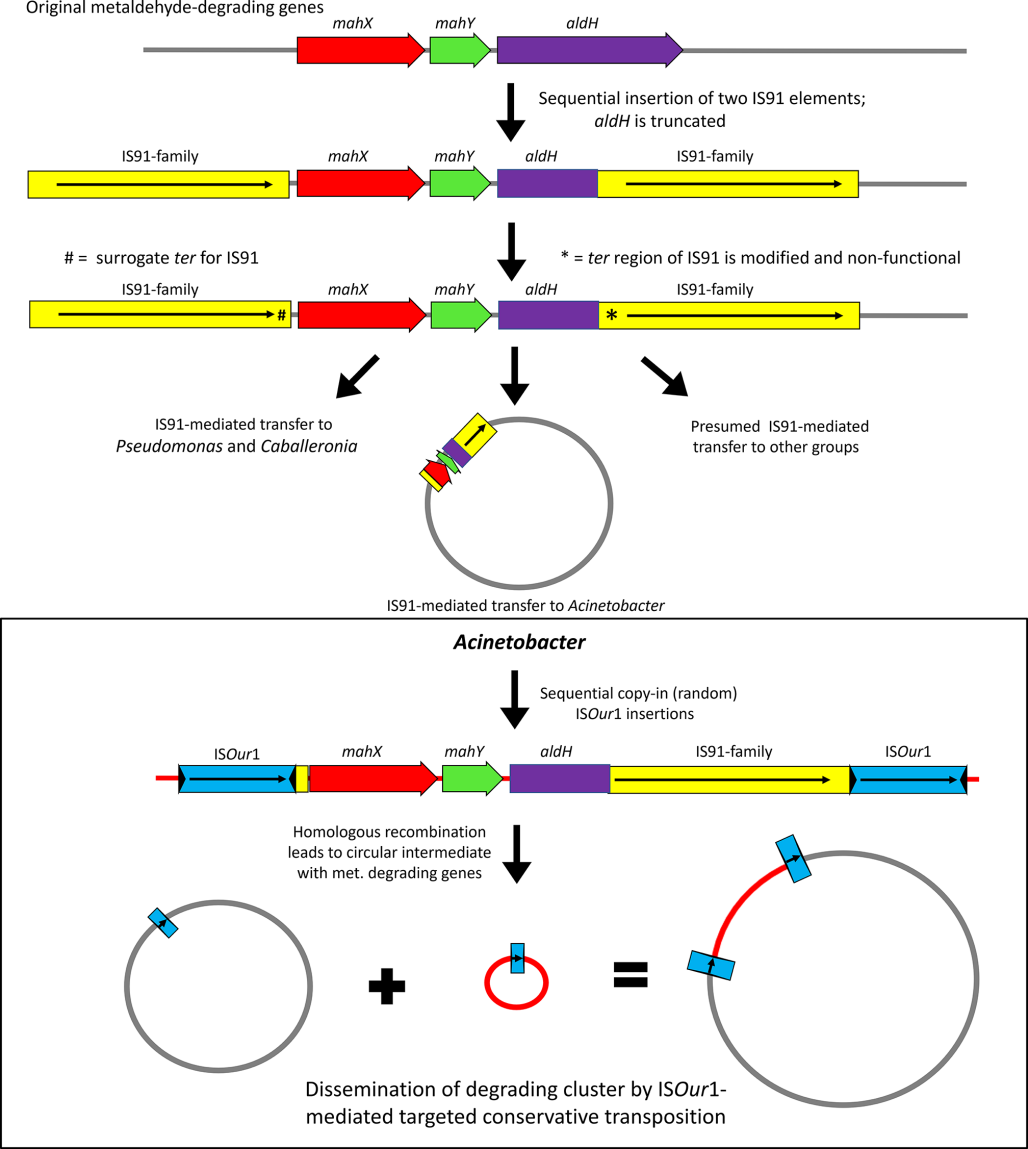
Possible evolutionary history leading to the metaldehyde-degrading gene cluster present in the isolated *Acinetobacter* strains.

As MahX had been discovered in a potential operon upstream from the predicted protein of MahY [7], examination of the *Sphingobium* sp. CMET-H genome was undertaken to identify potential similar sequences. Nevertheless, blast analysis of MahY resulted in no significant alignment matches. Neither an HMMER search against the Glyoxalase_4 HMM profile (PF13669.9), the pfam family to which MahY belongs, retrieved any hit from pSM1.

### MahS if responsible for the first step of metaldehyde degradation in in the *Sphingobium* CMET-H

To determine if MahS is the protein responsible for the first step of metaldehyde degradation in *Sphingobium* CMET-H, inducible heterologous expression of MBP-MahS was undertaken in *E. coli* BL21(DE3), and metaldehyde was added to the induced cultures. Adequate induction of MBP-MahS expression was corroborated using SDS-PAGE analysis (Fig. S5). Metaldehyde was depleted from 15 mg l^−1^ to below the limit of detection (0.05 mg l^−1^) following a 3.5 h incubation with induced *E. coli* BL21(DE3), while no removal was detected in the control BL21(DE3) culture containing the empty vector. Therefore, the *mahS* gene was determined to encode the protein responsible for the first step of metaldehyde degradation.

### Genetic context of the metaldehyde-degrading gene *mahS*

Examination of the genetic context of gene *mahS* within the *Sphingobium* CMET-H genome was undertaken. The *mahS* gene (921 bp) is located within the conjugative plasmid pSM1, between positions 51 299 and 52 219 (Fig. 3b). A gene coding for a protein with carboxymuconolactone decarboxylase-like domain (CMD), part of the AhpD-like homologous superfamily, is present immediately upstream. This family has been demonstrated to be involved in protocatechuate catabolism [61]. Further upstream, a cluster of six genes involved in the metabolism of the acetoin is present. Acetoin is an important metabolic product released by various microorganisms when growing on fermentable carbon sources through the Embden-Meyerhof pathway [62]. These genes include *acoA, acoB, acoC, acoD* (aldehyde dehydrogenase), *acoX* and *acsA* (acetyl-CoA synthetase). The *acoX* sequence, a predicted NAD kinase [63], is interrupted by an IS*21*-family insertion sequence, which is also found in reverse orientation downstream from *mahS*.

## Discussion

### Generation of reference-quality whole genome sequence of metaldehyde degrader *A. calcoaceticus* E1 and genetic context of the degrading genes in γ- and β-Proteobacteria

The plasmid responsible for metaldehyde degradation in strain E1, pAME76, shares with many other catabolic plasmids the characteristic of being a relatively large plasmid (>50 kb) [64]. It lacks the T4CP and T4SS components required to be self-transmissible by conjugation [65]. It also lacks the MOB relaxase and thus is also unable to be mobilized by a helper conjugative plasmid [66]. Nevertheless, the extensive similarity of the pAME76 plasmid backbone to the plasmids encoding metaldehyde-degrading genes found in *A. bohemicus* and *A. lwoffii* suggests horizontal transmission (Fig. S3). In fact, the latter plasmids were found to be potentially conjugative. Nevertheless, HGT not only relies on conjugation. Natural competence is characteristic to many environmental and clinical *Acinetobacter* strains [67], and specifically *A. calcoaceticus* is known to be transformed naturally at high efficiency [68, 69] so it is likely that plasmids which are non-mobile by conjugation could be acquired by natural transformation.

pAME76 harbours numerous ISs and transposons (24 complete or truncated transposase gene copies), including the two IS*Our*1 copies flanking the metaldehyde degrading genes and the IS*91*-like elements, possibly comprising a composite transposon. Catabolic genes are often flanked by insertion sequences, which may have facilitated gene acquisition by a specific replicon, but may also ease the subsequent exchange of the genes between different replicons and hosts [70, 71]. This transposon-like configuration is also found in the other degrading *Acinetobacter* strains, JMET-C and SMET-C, which suggests that it may have mediated the horizontal transfer of the metaldehyde-degrading genes between species in the genus.

IS*Our*1 belongs to the IS*6*/IS*26* family, currently comprising around 160 insertion sequences [72]. The full sequence of IS*6* has never been fully determined but it is thought to be identical or nearly identical to IS*26*, which has been the subject of most of the experimental studies in the family [73]. IS*26* has a very important role in the generation of genetic diversity in Gram-negative bacteria, and particularly relating to antibiotic resistance in *Acinetobacter* [74, 75]. IS*Our*1 itself has been implicated in the acquisition of cephalosporin resistance genes from *Acinetobacter* into the chromosome of *Oligella urethralis* [55]. Both IS*26* and IS*Our*1 are 820 bp long, with 14 bp inverted repeats and a single gene which encodes a 234 amino acid transposase [76]. IS*Our*1 is also very similar (>99 % nucleotide similarity) to IS*1008* (Genbank accession AJ251307, 6787–7606 bp). The mechanism of transposition of IS*Our*1 specifically has not been studied experimentally, however the details of this process in the flagship of the group, IS*26*, have been elucidated and other members of the family seem to share the same mechanism, suggesting that all members are able to use the same transposition routes [77].

IS*26* movement is different from most of the other families of insertion sequences and has been shown to be mediated by a transposase via two main routes: a copy-in route and a targeted conservative route [78]. The copy-in route involves two molecules, a donor molecule with an IS*26* copy and a target molecule lacking IS*26*. The resulting cointegrate contains two IS*26* copies, which flank the donor molecule as direct repeats. The cointegrate can in some cases be resolved by homologous recombination between the two directly oriented copies of the insertion sequence in a recombination proficient host, which would render a single IS*26* sequence surrounded by the 8 bp target site duplication in the target molecule, resembling a product of a simple transposition [76, 79, 80]. In the targeted conservative route both molecules carry a copy of IS*26* and also produce a cointegrate. It is targeted because it occurs at one or other end of the two insertion sequences instead of at a random site. It is conservative because the insertion sequence and the target site are not duplicated, no bases are lost or gained [78, 81]. IS*1008*, the closest related sequence to IS*Our*1, requires both identical transposases and identical DNA sequences at the reacting IS ends to carry out targeted integration [82], which is true in our case study.

Regarding the IS*91*-like elements these have been frequently observed in the vicinity of xenobiotic-degradation genes and have been implicated in their mobility [83]. Also, it has been suggested that IS*91* family elements might be hot spots for insertion of other transposable elements [83]. Sequence analysis has shown that the majority of IS*91* family isoforms do not appear in the classic way other insertion sequences do, as direct repeats flanking adjacent open reading frames forming composite transposons [83, 84]. Instead it has been shown that transposition of adjacent genes can occur through a ‘one-ended’ mechanism involving a single IS, which is translocated along with adjacent sequences of different lengths [83]. IS*91* uses a rolling circle mechanism for transposition [85] which in principle is initiated at the *ori* end of the insertion sequence (3′ to the transposase, where transposition starts) and terminates at the *ter* end (5′ to the transposase, where transposition ends). However, termination can fail in around 1–10 % of the events, and transposition is extended to neighbouring sequences, until a surrogate end sequence is reached [86, 87]; this can also happen if the *ter* region is deleted [88].

Given that in the *Acinetobacter* strains and in the *P. vancouverensis* SMET-B strain the *ter* region of the IS*91*-like insertion sequence is absent, we speculate that the neighbouring genes, the metaldehyde-degrading gene cluster, could have been transposed along with the IS*91*-like insertion sequence in this way, and afterwards the IS*Our*1-mediated transposition might have taken place for the *Acinetobacter* strains only, as suggested by the fact that the IS*91*-like sequence is interrupted by an intact IS*Our*1 sequence and not the opposite way. Nonetheless, the fact that IS*91* family elements do not generate target site duplications upon insertion [89] and IS*26* insertion sequences may or may not do it depending on the specific transposition mechanism they use [78] it is difficult to trace the specific evolutionary history of the cluster. Even so, one might speculate on the possible succession of events to generate the typical cluster observed within the isolated *Acinetobacter* strains (Fig. 5).

The presence of the IS*91* and IS*26* family elements in the cluster and the different GC nucleotide percentages between the gene cluster (59.81–62.74 %) and the whole genome sequences of metaldehyde-degrading *Acinetobacter* strains (38.7–40.2 %) are parametric indicators of HGT [56]. These observations along with the fact that the plasmids in several of the strains share extensive regions (Fig. S3) reveal that HGT of the degrading cluster has occurred.

### Identification of the gene responsible for the first step of metaldehyde degradation in *Sphingobium* CMET-H and examination of its genetic context

Metaldehyde degrading genes that had been identified previously were present in the *mahX-mahY-aldH* cluster shared within γ- and β-Proteobacteria [7]. Sequence homology analyses using *mahX* as query, pointed towards *mahS*, a closely related dioxygenase family gene, as the main candidate gene encoding for the initial step of metaldehyde degradation in *Sphingobium* CMET-H (α-Proteobacteria). Experimental verification revealed that the MahS protein confers the ability to degrade metaldehyde upon heterologous expression in *E. coli *.

Regarding the genetic context of the *mahS* gene, it is found immediately next to a gene encoding a protein with a CMD domain, located closely to genes involved in acetoin metabolism and to insertion sequences. Contrary to the clear role of the IS elements for dissemination of the *mahX-mahY-aldH* gene cluster in *Acinetobacter* and other taxa, in this case, a direct involvement of the IS*1*-family in the acquisition and dissemination of *mahS* is not immediately evident. Since *Sphingobium* CMET-H is the only α-Proteobacteria isolated so far that has been shown to be capable of metaldehyde degradation, no comparative analysis with other strains can be performed. Even though two IS*1*-family elements are present in close proximity, it is unlikely that they form a composite transposon with the genes they flank, because the internal and external genes are all related with acetoin metabolism. Therefore, the most likely explanation is that this insertion sequence has translocated itself, without accompanying genes.

The *mahS* gene, as a close homolog to *mahX*, would encode for an enzyme which would catalyse an identical reaction on the metaldehyde molecule, starting its degradation by oxygenation and ring cleavage, generating the predicted product 1,3,5,7-tetramethyl-2,4,6-trioxa-1-hydroxy-7-octanone, a hemiacetal. In the original degradation pathway [7] it was proposed that a lyase encoded by *mahY* would then accelerate the iterative breakdown of the hemiacetal intermediate to acetaldehyde. However, no homologs to *mahY*, the gene encoding the following enzyme in the original metaldehyde degradation pathway, were found in the *Sphingobium* CMET-H genome. Consequently, the pSM1 plasmid proteome was manually inspected for lyases that could potentially catalyse this step, the only possible candidate being the CMD-encoding gene located next to *mahS*. Nevertheless, the cognate substrate for this family of enzymes, carboxymuconolactone, is not a hemiacetal, and thus, its involvement in the metaldehyde degradation pathway of *Sphingobium* CMET-H is speculative and would have to be corroborated experimentally. Until then, this remains an open question.

Genes involved in acetoin metabolism are located upstream from *mahS*. Besides being a metabolic product, acetoin can also be degraded and used as a carbon and energy source when other more easily assimilable molecules are depleted [90]. Acetoin biodegradation has been shown to be catalysed in numerous bacteria by the acetoin dehydrogenase enzyme system (*acoA*, *acoB*, *acoC*), yielding acetyl-CoA and acetaldehyde [91]. Acetaldehyde is then oxidised to acetate by an aldehyde dehydrogenase (*AcoD*). The resulting acetate in converted to acetyl-CoA by the acetyl-CoA synthetase (*acsA*), and then enters central carbon metabolism [92, 93]. Interestingly, acetaldehyde is also the main intermediate degradation product of biotic metaldehyde degradation [94]. Either as a product of acetoin or metaldehyde degradation, acetaldehyde could be further metabolised by the enzymes in the acetoin catabolic pathway. AcoD transforms acetaldehyde into acetate, while the action of AcsA produces acetyl-CoA, which can be directly incorporated into central metabolism.

Hence, we speculate that pSM1 might be able to provide a suitable host with several of the accessory genes needed for metaldehyde catabolism. Dissemination of the metaldehyde-degrading genes would be facilitated by the fact that pSM1 encodes the full set of genes needed for conjugative transfer. Therefore, as with the *mahX-mahY-aldH* cluster, it appears that a MGE, in this case a conjugative plasmid, would be responsible for the dissemination of the degrading genes to other hosts. This hypothesis would be validated if further studies find metaldehyde-degrading organisms with this genetic determinant as well. Simultaneously, experimental validation of the pSM1 conjugative capabilities, preferably to a naïve host (neither having metaldehyde nor acetoin degrading pathways) would further clarify the dispersal of metaldehyde degrading capabilities.

## Conclusions

Bioremediation strategies to deal with metaldehyde in drinking water have shown encouraging results, however its biological degradation mechanisms and how they are dispersed amongst prokaryotes are still not completely understood. Here, we have described in detail the genetic platforms which mediate this process, which include insertion sequences, transposons, non-conjugative and conjugative plasmids. Moreover, the involvement of the *mahS* gene in the first step of metaldehyde biodegradation in the biotechnologically-relevant strain *Sphingobium* CMET-H has been verified experimentally and insights into a possible degrading pathway have been presented. This increases our understanding on the behaviour of xenobiotic-degrading traits in prokaryotic communities and will aid in the design of bioaugmentation strategies for drinking water treatment technologies for metaldehyde in the near future.

## Supplementary Data

Supplementary material 1
