# Supplementary material for: Dissemination of metaldehyde catabolic pathways is driven by mobile genetic elements in Proteobacteria
Source: Microb Genom. 2022 Oct 27;8(10):mgen000881. doi: 10.1099/mgen.0.000881 (PMC9676059; doi:10.1099/mgen.0.000881)
Supplement: Supplementary material 1 [file mgen-8-881-s001.pdf]

Supplementary Table S1. Oligonucleotides used in this study.

Primer name	Sequence 5' - 3'	Purpose
pETFPP F	CGCGCCTTCTCCTCACATATGGCTAGC	Amplification of pETFPP
pETFPP R	TTGCTGGTCCCTGGAACAGAACTTCC	Amplification of pETFPP
mbp-mahS R	TGAGGAGAAGGCGCGTCAGGCTTCCAGGCTCACC	Amplification of mbp-mahS
mbp-mahS F	TCCAGGGACCAGCAATGTCCGAGGTCGACACCCT	Amplification of mbp-mahS

Supplementary Table S2. Quality statistics for Illumina short read whole-genome sequencing run and assembly of metaldehyde-degrading strains.

	A. calcoaceticus **E1**	***Sphingobium*** **CMET-H**
**Median insert size (bp)**	321	671
**Mean coverage (fold)**	70.3	68.3
**Number of reads**	774 073	818 547
**Number of contigs**	112	91
**Largest contig (bp)**	377 626	644 866
**Total length (bp)**	4 402 996	5 650 245
**GC (%)**	38.7	63.6
**N50 (bp)**	105 976	220 888
**L50 (bp)**	13	7
**# N's**	0	0

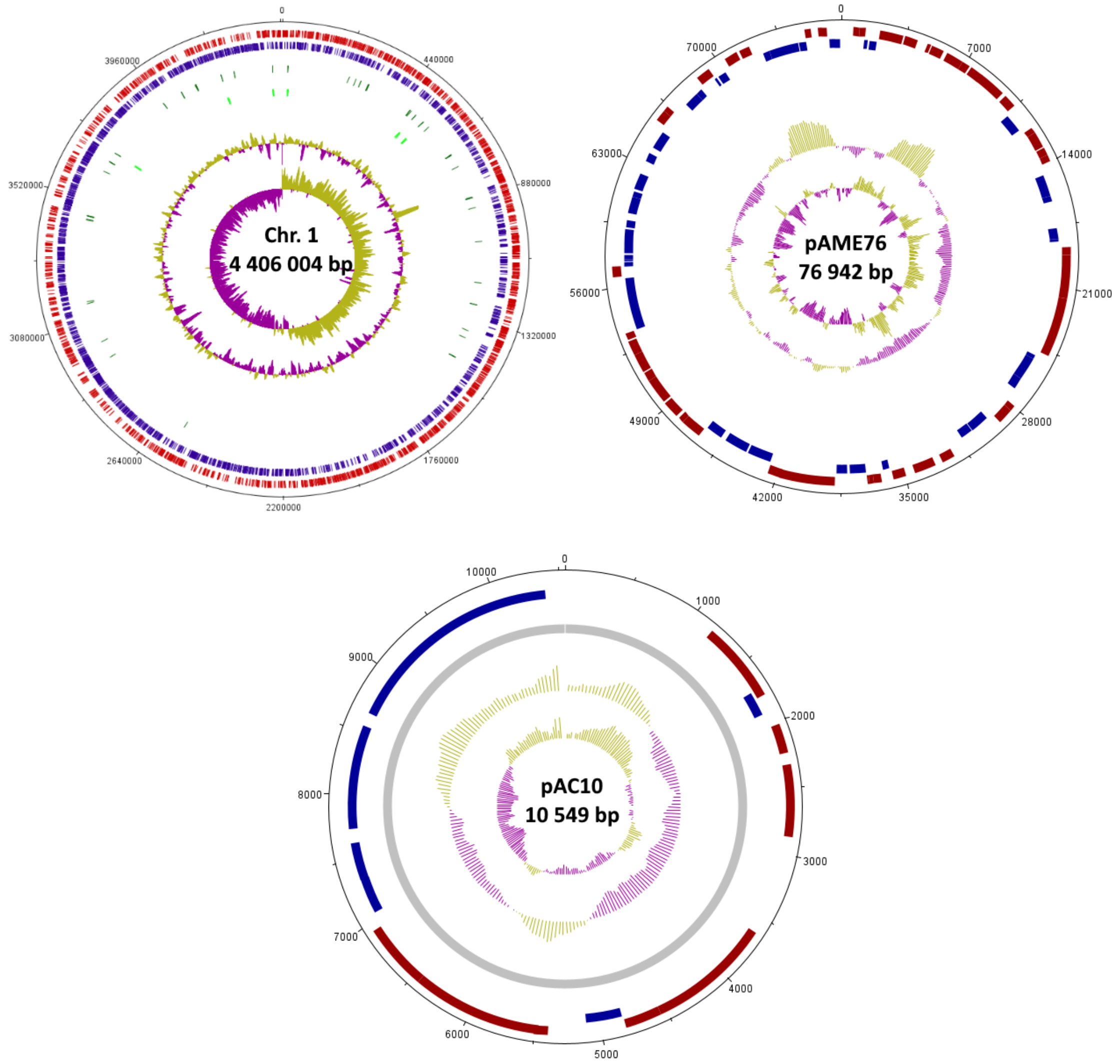

Supplementary Figure S1. *A. calcoaceticus* E1 replicon map. Sequence plots were generated using Artemis and DNAPlotter [1, 2]. From outside to inside: open reading frames for the forward and reverse strands in red and blue; tRNA genes in dark green; rRNA genes in light green; GC content (purple: below average, gold: above average); GC skew (purple: below average, gold: above average).

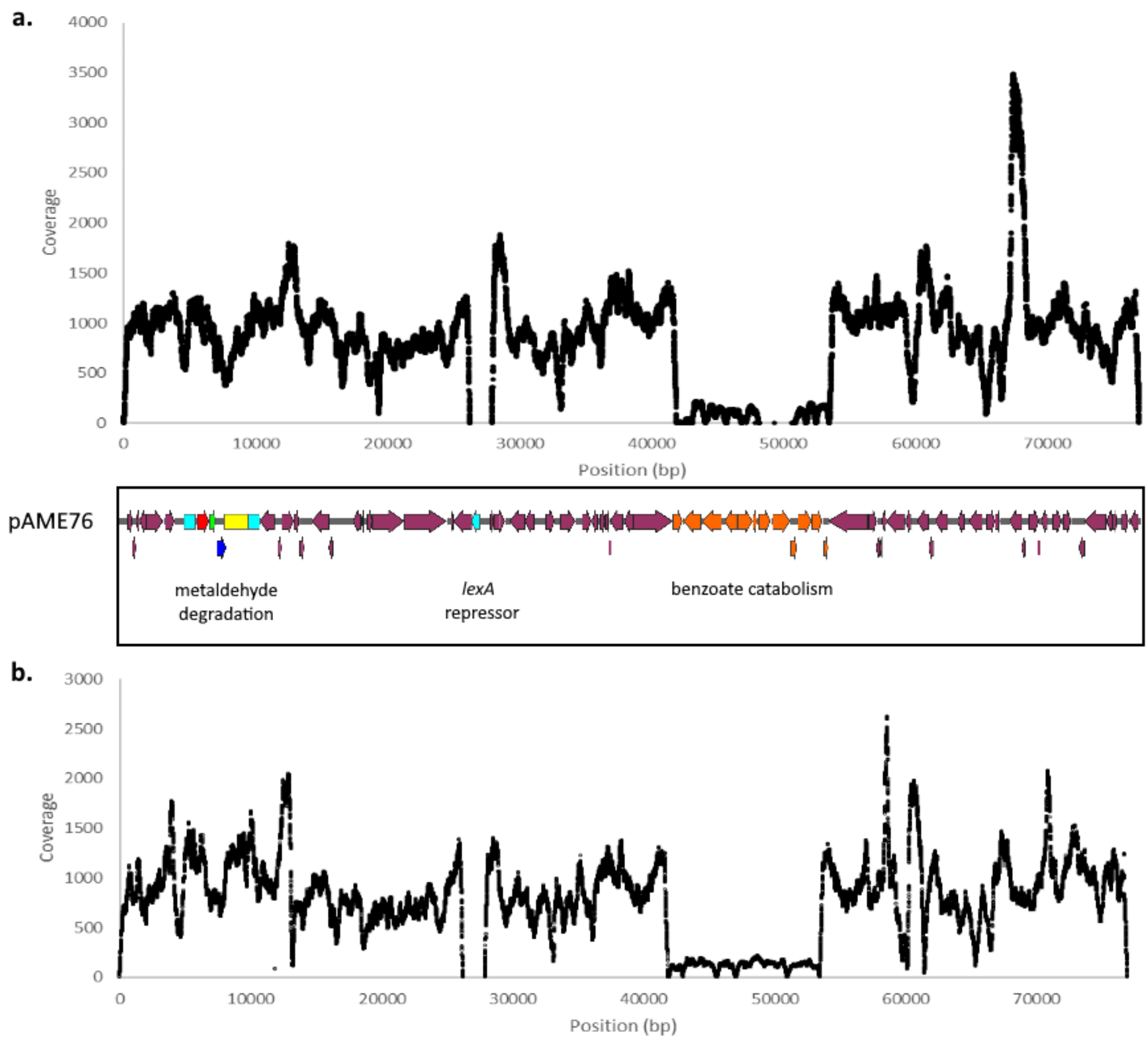

Supplementary Figure S2. Coverage resulting of mapping the reads from **a.** *A. bohemicus* JMET-C and **b.** *A. lwoffii* SMET-C whole-genome sequencing against pAME76 plasmid reference using Burrows-Wheeler Alignment tool (BWA) [3] in UGENE [4, 5].

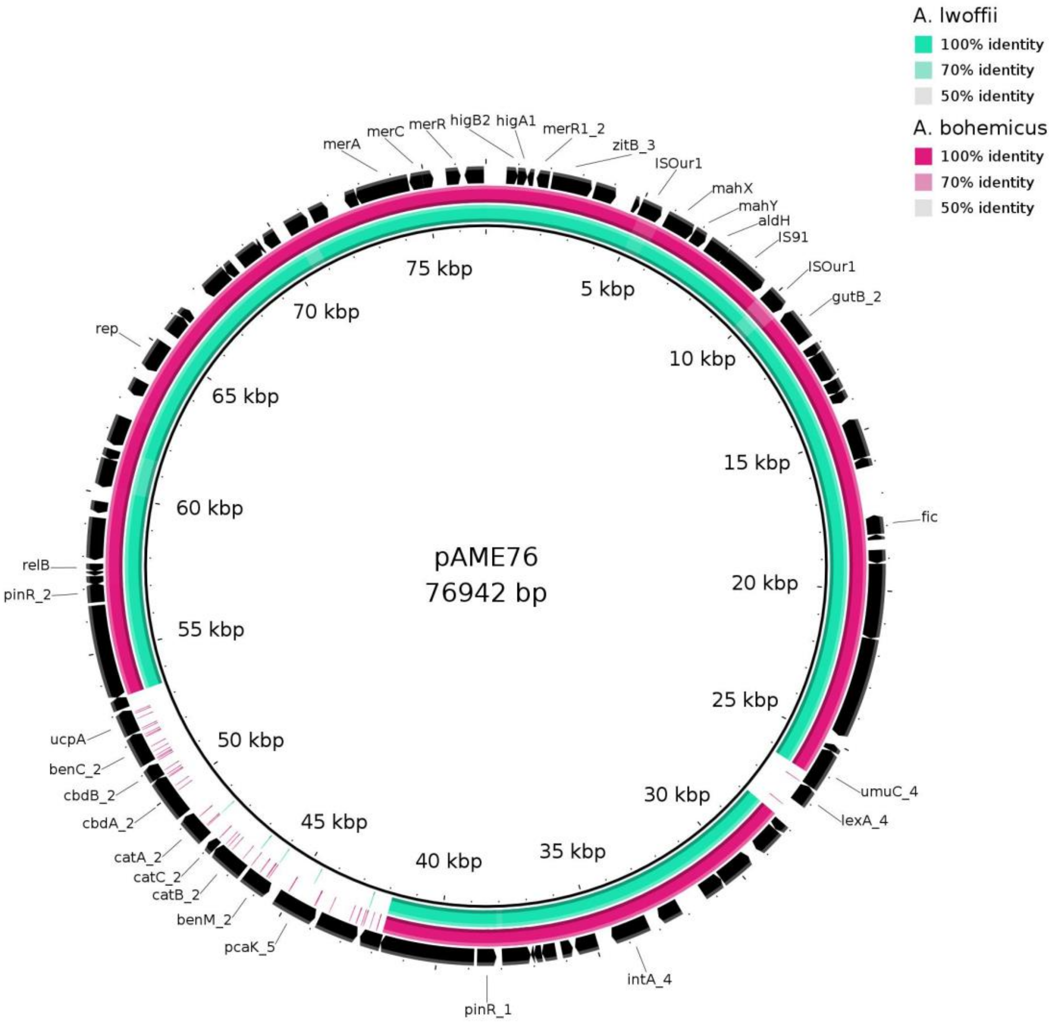

Supplementary Figure S3. Genomic comparison of the pAME76 plasmid of *A. calcoaceticus* (reference) with plasmids from metaldehyde-degrading isolates of *A. lwoffii* and *A. bohemicus* (colored rings). Comparisons were performed with BRIG (Alikhan, N. F., Petty, N. K., Ben Zakour, N. L., & Beatson, S. A. (2011). BLAST Ring Image Generator (BRIG): simple prokaryote genome comparisons. *BMC genomics*, *12*, 402. https://doi.org/10.1186/1471-2164-12-402)

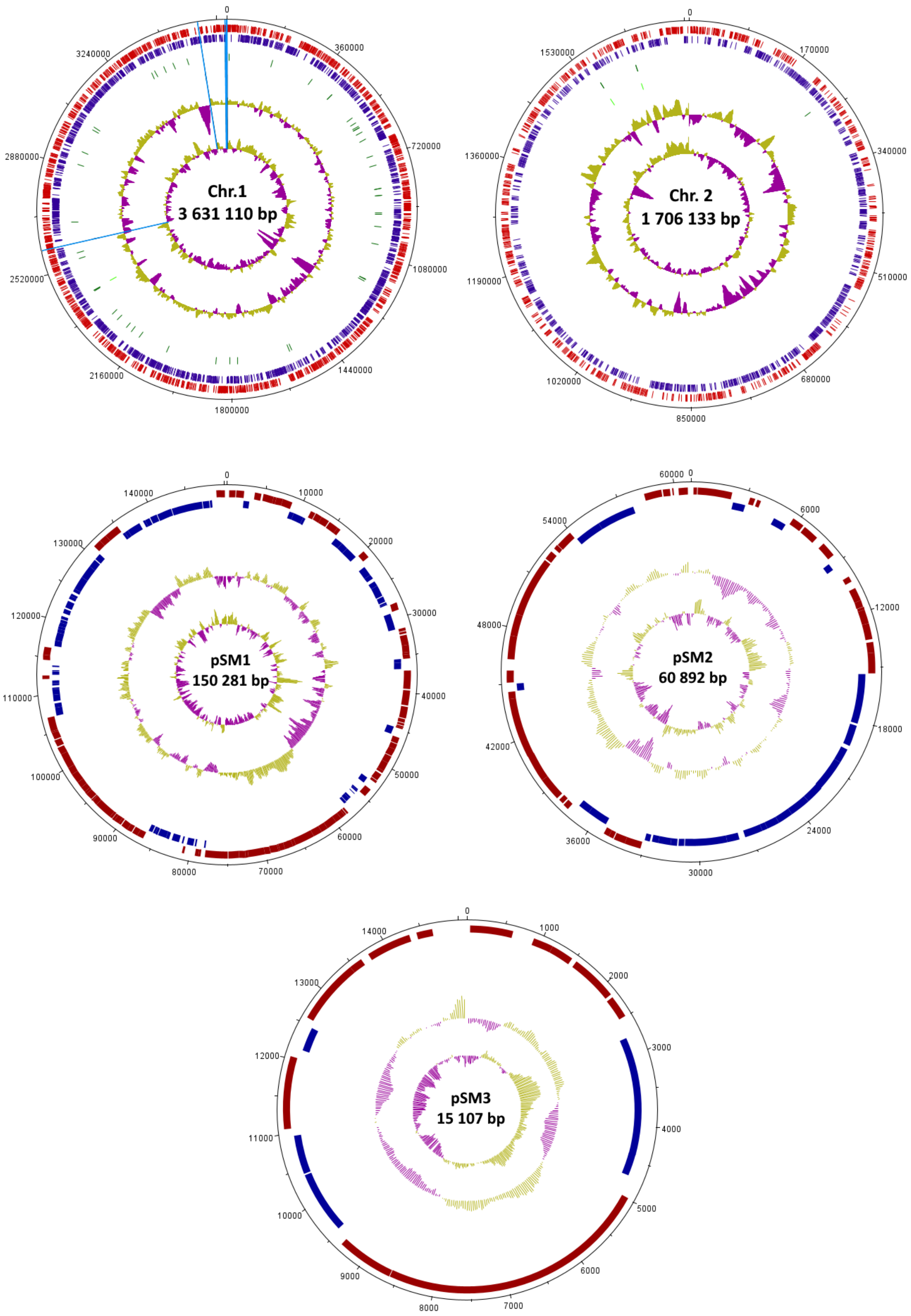

Supplementary Figure S4. *Sphingobium* CMET-H replicon map. From outside to inside: open reading frames for the forward and reverse strands in red and blue; tRNA genes in dark green; rRNA genes in light green; GC content (purple: below average, gold: above average); GC skew (purple: below average, gold: above average). Different contigs in the same molecule are separated by light blue lines.

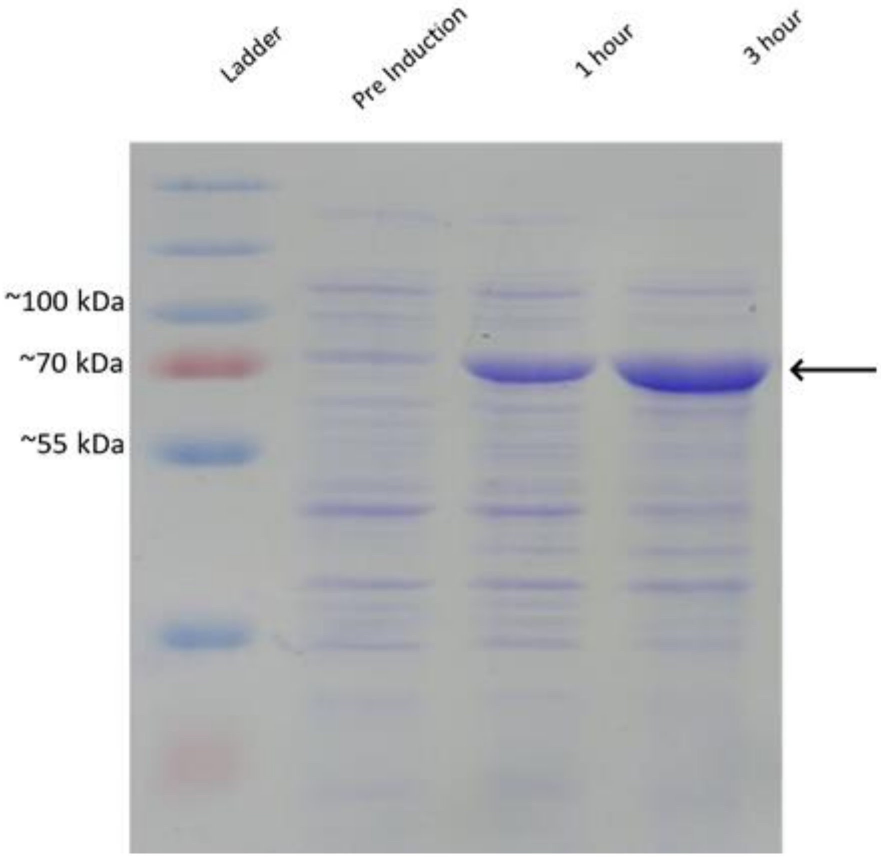

Supplementary Figure S5. 10% SDS PAGE gel with samples from pre-induced culture with, 1 h post IPTG induction and 3 h post IPTG induction of MBP-MahS expressing *E. coli* BL21. Pageruler was used to determine approximate size. 15 µL of sample were loaded to each well. The predicted protein fusion was expected to be ~78 kDa (arrow).

Supplementary Methods - Heterologous expression of *mahS*

For identification of the metaldehyde degrading gene within *Sphingobium* sp. CMET-H, the candidate gene *mahS* was inserted into the *E. coli* expression vector pETFPP_2 [6]. Amplification of both the insert gene and vector was performed using the High-Fidelity Phusion Polymerase (Thermo Fisher Scientific), following the manufacturer's protocol. Primers for *mahS* and pETFPP_2 amplification were designed using Takara's primer design tool. Ligation of the product to the insert region was performed through isothermal assembly using a NEBuilder HiFI DNA assembly kit following the manufacturer's protocol. Following transformation, kanamycin-resistant colonies were screened through PCR amplification and gel analysis. Transformants identified to possess the appropriate gene insert were verified through Sanger sequencing. Upon identification of the correct insert, the construct was used to transform *E. coli* BL21 (DE3).

Protein induction was performed by incubating 50 mL of LB supplemented with 50 µg/mL kanamycin with 500 µL of overnight culture. Samples were grown for ~ 3 hours until OD_{600} of 0.4-0.6 was achieved. Where induction was required, isopropyl β- d-1-thiogalactopyranoside (IPTG) (Sigma-Aldrich) was added to ensure a final concentration of 1 mM. Samples were then incubated at 30°C whilst shaking at 220 rpm. Following growth, cell OD_{600} was calculated, and the cells normalised to an OD_{600} of 1.0 for a final volume of 10 mL. Samples were transferred to a sterile 50 mL Falcon tube and centrifuged for 10 mins at 6,000 g and the pellet washed in 10 mL of Minimal Salts Medium. Following this, cells were centrifuged again for 10 mins at 6,000g and the pellet resuspended into 10 mL of MSM supplemented with metaldehyde (15 mg L^{-1}). Samples were then incubated at 30°C at 180 rpm for 3.5 hours. All assays were performed in triplicate. Following incubation, 1mL was extracted from each sample for metaldehyde quantification. To remove cell debris and other particulate matter, samples were centrifuged for 10 mins at 4000 g. From the supernatant, 400 µL was extracted and mixed with 500 µL of dichloromethane within a glass chromatography vial. Samples were then vortexed for 30 sec and allowed to settle for 30 mins. From the lower organic phase, 5 µL was extracted and injected into the inlet of an Agilent 7820A gas chromatograph. To observe protein size and presence, SDS-PAGE

gels were used [7]. Cell lysate was prepared through the addition of loading buffer to sample pellet in a 1:1 mixture. This mixture was then heated to 98oC for 5 minutes using a heat block.

Supplementary Results – Technical description of the *A. calcoaceticus* E1 genome

Chromosome

The map of the 4.4 Mb chromosome is displayed on Figure 1. Automated annotation identified 4292 coding sequences (CDSs) in the main chromosome. The starting point for the chromosome sequence was chosen to be the first base pair of the chromosomal replication initiator protein (*dnaA*). A 682 bp origin of replication (*oriC*) region was located immediately preceding the start codon of *dnaA* and showed a 98.2% sequence similarity to the *oriC* locus region of *A. calcoaceticus* strain CA16 (ORI97013474) from the DoriC replication origin database [8]. The location of the *oriC* region clearly matches the sign change in the GC skew calculation (Figure 1), which also provides an approximate estimation of the position of the replication terminus at the opposite end of the chromosome. Six rRNA operons were identified using RNAmmer [9], which is consistent with the mean for *Acinetobacter baumanni/calcoaceticus* genomes in rrnDB [10], while 75 tRNA genes were detected using tRNAscan-SE [11].

Plasmid pAME76

Automated annotation identified 81 CDSs in the 76.9 kb circular low copy-number plasmid (PCN=2.8), from here on called pAME76, of which 24 are transposases, 12 are hypothetical proteins with no annotation, 10 are involved in benzoate compound catabolism, 6 are part of toxin-antitoxin plasmid stability systems, 6 are related to mercuric resistance, 5 are integrases or recombinases, 3 are the metaldehyde-degrading proteins (including *aldH*), 2 are cation transporters, 1 encodes a replication initiation protein of the Rep_3 superfamily (PF01051), 1 is a DNA polymerase and 11 encode for various other functions. The plasmid map is shown in Figure 1. No *oriT* region, relaxase gene, T4CP and T4SS components were found which implies that pAME76 is non-transmissible by conjugation. pAME76 is a low copy-number plasmid (2.8 copies per chromosome).

Plasmid pAC10

An additional 10.5 kb circular high copy-number plasmid (PCN=494) is present in *A. calcoaceticus* E1, pAC10 (Figure 1). It encodes 13 CDSs, including a replication initiator protein of the Rep_3 superfamily, a relaxase, a transposase, a resolvase, two proteins involved in toxin-antitoxin plasmid stability, three hypothetical proteins with no annotation and four genes encode for other various other functions. No *oriT* was predicted but the MOB_Q class relaxase that shares 99.1% identity with that from *Acinetobacter haemolyticus* (WP_161412392.1) was found. No MPF genes are encoded, therefore this is a putative mobilizable plasmid. No plasmids highly similar to pAC10 are recorded in the RefSeq database, as indicated by COPLA results.

Supplementary Results – Technical description of the *Sphingobium* CMET-H genome

Chromosomes

The maps of the 3.6 Mb chromosome 1 (Chr. 1) and the 1.7 Mb chromosome 2 (Chr. 2) are displayed in Supplementary Figure S4. Automated annotation identified 3512 CDSs for the former and 1468 CDSs for the latter. The protein sequences for the chromosomal replication initiator protein (DnaA) from Chr. 1 and Chr. 2 share 99.8% and 98.5% sequence similarity to the ones from *Sphingobium* sp. 15-1 (WP_176594952 and WP_176598516.1). Three rRNA operons were identified (one in Chr. 1 and two in Chr. 2), which is consistent with the mean for *Sphingobium* genomes in rrnDB [10]. 55 tRNA genes (51 in Chr. 1 and 4 in Chr. 2) were also identified.

Plasmid pSM1

PROKKA annotation identified 163 CDSs in the 150.3 kb circular low copy-number plasmid pSM1 (PCN=1.49) (Figure 4a), of which 32 are related with conjugative transfer, including a MOB_P class relaxase, a T4CP and two T4SSs corresponding to the MPF_T and MPF_F types, which indicates that pSM1 is a conjugative plasmid. Other CDSs identified encoded 10 transposases or inactive derivatives, 7 related to acetoin metabolism, 7 involved in plasmid replication and partitioning (including a Rep_3 replication initiation protein), 3 belonging to toxin-antitoxin plasmid stability systems, 1 phytanoyl-CoA dioxygenase family protein, 25 hypothetical proteins with no annotation, and the rest encode for various other functions.

Plasmid pSM2

A 60.9 kb circular low copy number plasmid (PCN=1.67) encoding for 67 CDSs was found to be part of the *Sphingobium* CMET-H genome. 13 CDSs are related with conjugative transfer (including a MOB_P relaxase, a T4CPand a T4SS belonging to the MPF_T type), 5 are involved in resistance to metals, 3 relate to plasmid replication and partitioning (including a Rep_3 replication initiation protein), 2 are part of toxin-antitoxin plasmid stability systems, 9 are hypothetical proteins with no annotation, and the rest

encode for various other functions. No *oriT* was predicted, probably due to a limitation of the oriTFinder database.

Plasmid pSM3

A 15.1 kb circular low copy number plasmid (PCN=1.49) encoding for 18 CDSs was also found to be part of the *Sphingobium* CMET-H genome. CDSs include a putative replication initiation protein, MOB_Q relaxase, a T4CP, 2 proteins involved in toxin-antitoxin plasmid stability, 2 hypothetical proteins with no annotation and 11 genes encode for other various other functions. No *oriT*, nor T4SS components were predicted, indicating that pSM3 is a putative mobilizable plasmid.

References

1. **Carver T, Thomson N, Bleasby A, Berriman M, Parkhill J**. DNAPlotter: circular and linear interactive genome visualization. *Bioinformatics* 2009;25:119–120.
2. **Carver T, Harris SR, Berriman M, Parkhill J, Mcquillan JA**. Artemis: an integrated platform for visualization and analysis of high-throughput sequence-based experimental data. *Bioinformatics* 2012;28:464–469.
3. **Li H, Durbin R**. Fast and accurate short read alignment with Burrows-Wheeler transform. *Bioinformatics* 2009;25:1754–1760.
4. **Okonechnikov K, Golosova O, Fursov M, UGENE team**. Unipro UGENE: a unified bioinformatics toolkit. *Bioinformatics* 2012;28:1166–7.
5. **Golosova O, Henderson R, Vaskin Y, Gabrielian A, Grekhov G, *et al.*** Unipro UGENE NGS pipelines and components for variant calling, RNA-seq and ChIP-seq data analyses. *PeerJ* 2014;2:e644.
6. **Fogg MJ, Wilkinson AJ**. Higher-throughput approaches to crystallization and crystal structure determination. *Biochem Soc Trans* 2008;36:771–775.
7. **Cold Spring Harbor**. SDS-PAGE Gel. *Cold Spring Harb Protoc* 2015;2015:pdb.rec087908.
8. **Luo H, Gao F**. DoriC 10.0: an updated database of replication origins in prokaryotic genomes including chromosomes and plasmids. *Nucleic Acids Res* 2019;47:D74–D77.
9. **Lagesen K, Hallin P, Rødland A, Staerfeldt H-H, Rognes T, *et al.*** RNAmmer: consistent and rapid annotation of ribosomal RNA genes. *Nucleic Acids Res* 2007;35:3100–3108.
10. **Stoddard SF, Smith BJ, Hein R, Roller BRK, Schmidt TM**. rrnDB: improved tools for interpreting rRNA gene abundance in bacteria and archaea and a new foundation for future development. *Nucleic Acids Res* 2015;43:D593-8.
11. **Lowe TM, Chan PP**. tRNAscan-SE On-line: integrating search and context for analysis of transfer RNA genes. *Nucleic Acids Res* 2016;44:W54–W57.
